# Increasing Role of Roof Gutters as *Aedes aegypti* (Diptera: Culicidae) Breeding Sites in Guadeloupe (French West Indies) and Consequences on Dengue Transmission and Vector Control

**DOI:** 10.1155/2012/249524

**Published:** 2012-04-02

**Authors:** Joël Gustave, Florence Fouque, Sylvie Cassadou, Lucie Leon, Gabriel Anicet, Cédric Ramdini, Fabrice Sonor

**Affiliations:** ^1^Service de Lutte Anti-vectorielle, Agence Régionale de la Santé de Guadeloupe, Le Raizet, 97110 Les Abymes, Guadeloupe; ^2^Institut Pasteur de la Guadeloupe, Laboratoire d'Entomologie Médicale, Morne Jolivière, BP484, 97183 Les Abymes, Guadeloupe; ^3^Cellule Inter Régionale d'Epidémiologie des Antilles et de la Guyane (CIRE), Agence Régionale de Santé de Guadeloupe, Bisdary, Rue des Archives, 97113 Gourbeyre, Guadeloupe

## Abstract

During the past ten years, the islands of Guadeloupe (French West Indies) are facing dengue epidemics with increasing numbers of cases and fatal occurrences. The vector *Aedes aegypti* is submitted to intensive control, with little effect on mosquito populations. The hypothesis that important *Ae. aegypti* breeding sites are not controlled is investigated herein. For that purpose, the roof gutters of 123 houses were systematically investigated, and the percentage of gutters positive for *Ae. aegypti* varied from 17.2% to 37.5%, from humid to dry locations. In the dryer location, most of houses had no other breeding sites. The results show that roof gutters are becoming the most important *Ae. aegypti* breeding sites in some locations in Guadeloupe, with consequences on dengue transmission and vector control.

Dengue fever (DF) is the most important arthropod-borne transmitted disease worldwide with 50 to 100 million DF infections, 500,000 dengue hemorrhagic fever (DHF) hospitalized cases, and 20,000 to 25,000 deaths [1]. In the Americas, DF and DHF are endemic, and in 2010 more than 50 countries reported 1,663,276 clinical DF cases, 48,954 DHF cases, 1,194 deaths, and the cocirculation of the four dengue serotypes [2]. Though suspected to be present since a long time, dengue was first detected in Guadeloupe (French West Indies) around the 80s [3]. The disease was considered rare until the first DEN-2 epidemic in 1992–1994 [4] and the first report of DHF cases in 1995 [5]. The last epidemic started at the end of 2009 and continued all 2010 with about 40,000 clinical DF cases, 418 severe DF cases, and 5 deaths [6]. This epidemic was the largest one reported from Guadeloupe. Dengue viruses are transmitted by the mosquitoes *Aedes aegypti* L. 1762, and because no dengue vaccine is available, the control of *Ae. aegypti* populations is the only tool to avoid further DF and DHF epidemics. Until recently, the main *Ae. aegypti* breeding sites in Guadeloupe were the 200 liters drums in urban and periurban situations [4]. The roof gutters were reported as *Ae. aegypti* breeding sites since a long time, but their mosquito production was considered negligible compared to other ground breeding sites [7]. Consequently roof gutters are often neglected in the routine survey of the mosquitoes breeding sites and are not included in the estimation of the Breteau Index [8]. However, an unpublished survey carried out in Guadeloupe in 1998 found that about 30% of roof gutters were positive for *Ae. aegypti* and could produce huge numbers of mosquitoes. Roof gutters are still functioning when all accessible breeding sites have been controlled and may represent a reservoir for *Ae. aegypti*. To better estimate the importance of roof gutters as breeding sites for *Ae. aegypti* populations in Guadeloupe, a survey was carried out in 2006 in 3 different environmental areas of Guadeloupe.

The groups of houses investigated were chosen in residential areas situated in different climatic zones of Guadeloupe: (i) in the city of Saint-François situated at the eastern extremity of Grande-Terre (hot and dry climate, annual rainfall 1,300 mm, and mean annual temperature 25.6°C), (ii) in the village of Saint-Claude situated in the inland of Basse-Terre (cool and humid climate, annual rainfall 4,463 mm, and mean annual temperature 24.2°C), (iii) in the city of Baie-Mahault situated in the northern part of Basse-Terre (intermediate climate, mean annual rainfall 1,679 mm, and mean annual temperature 26.4°C). The climatic data were provided by the French meteorological services. For each location, 40 houses (representing about 10% of the 4,000 houses reported for each city) were selected randomly to have their roof gutters investigated in January, February, and May 2006 in Saint-François, Baie-Mahault, and Saint-Claude, respectively. The presence of water and the mosquito abundances (larvae and pupae) in the roof gutters were estimated by direct observation, and samples were collected for mosquito identification. The height, length, form and material of the roof gutter, and the presence of vegetal elements, detritus, and/or sediments were also reported. The mosquitoes were kept until adult emergence for their species identification. The relation between *Ae. aegypti* larvae, and pupae presence and other water parameters were analyzed with the Fisher exact test.

 Among the 123 houses investigated, representing 520 m of roof gutters, 81 (65.8%) contained water, 35 (28.4%) were positive for *Ae. aegypti* larvae and 20 (16.3%) were positive for *Ae. aegypti* pupae. All positive roof gutters were breeding *Ae. aegypti* individuals. *Culex *sp. larvae were found breeding with *Ae. aegypti* in only one gutter. Larvae of *Ceratopogonidae* of the genus *Dasyhelea* were also observed in several roof gutters. The abundance of *Ae. aegypti* larvae and pupae in roof gutters was highly variable, and important differences were observed between the studied sites, with a high percentage of positive roof gutters in Saint-François: 67% positive for larvae and 37% positive for pupae and a lower percentage in Saint-Claude: 27% positive for larvae and 17% positive for pupae. The dryer study site of Saint-François had the highest percentage of roof gutters positive for *Ae. aegypti* larvae and pupae, the location of Baie-Mahault with intermediate climate had the intermediate value, and the more humid study site of Saint-Claude had the lowest percentage of positive roof gutters. This finding was in agreement with previous observations (unpublished data) showing that roof gutters could serve as breeding “reservoirs” for *Ae. aegypti* in the dryer locations in Guadeloupe. The highest numbers of *Ae. aegypti* larvae and pupae were found in roof gutters containing water with sediments and water with vegetal detritus. The exact test of Fisher estimates show that the presence of *Ae. aegypti* pupae in roof gutters was significantly different between roof gutters with “clean” water and roof gutters with sediments and/or vegetal detritus (*P* = 0.0305, OR = 8.8 for sediments, *P* = 0.0135, OR = 11 for vegetal detritus). For *Ae. aegypti* larvae no significant difference was found, but the presence of sediments and/or vegetal was reported with the highest abundance of *Ae. aegypti* larvae (Figure 1). For densities higher than 100 larvae/liter, the percentage of positive roof gutters containing sediments and/or vegetal detritus was about three to four times higher than the percentage of positive roof gutters with “clean” water. This finding indicates that *Ae. aegypti* abundance is increasing with the presence of sediments and/or vegetal detritus (Figure 1), and on the contrary the absence of *Ae. aegypti* larvae is found more often in “clean” waters. The mean size of the positive roof gutters was about 6 m per house.

The main conclusion of this survey is the increasing importance of roof gutters as *Ae. aegypti* breeding sites in some locations in Guadeloupe. This type of breeding sites though recognized since about 50 years [7] is often neglected in the control operations due to the difficulty of access and the low mosquito production. But, in Guadeloupe the situation is changing, although the survey was made during the dry season (from January to May), about two-third of the roof gutters contain waters and 44% were positive for *Ae. aegypti* larvae. This type of containers was thus representing kilometers of *Ae. aegypti* breeding sites. These results are in agreement with studies done in Australia where the production of* Ae. aegypti* by roof gutters was estimated to account for 52.6% and 39.5% of the total population during wet and dry seasons, respectively, in urban areas [9]. In the Americas, the oviposition of *Ae. aegypti* in gutters surrounding high-elevated apartments has also been reported with consequences on dengue transmission [10]. Unexpectedly, the highest percentage of positive roof gutters was found in the driest area of Saint-François. This town was an important dengue focus during the 2006-2007 and 2009–2011 epidemics, but this town has one of the lower Breteau Index of Guadeloupe. Our results show that the *Ae. aegypti* densities in Saint-François are probably strongly underestimated, due to the absence of roof gutters investigations during routine surveillance. It thus appears necessary to systematically include roof gutters in the estimation of the *Ae. aegypti* indices. Following the present survey, the roof gutters Breteau indices of Saint-François, Baie-Mahault, and Saint-Claude were 40, 27, and 20, respectively. Furthermore, the positive roof gutters must be treated as any other *Ae. aegypti* breeding site. Another conclusion of the survey was the association between the presence of sediments and/or vegetal detritus and the presence of *Ae. aegypti* in the roof gutters. The presence of vegetal detritus was significantly associated with the highest *Ae. aegypti* pupae densities pointing out the role of the roof gutters environment and exposition.

Finally, the increasing role of roof gutters as *Ae. aegypti* breeding sites, demonstrated in this study, has consequences on dengue transmission and prevention. Firstly, recommendations on the roof gutters construction (correct water evacuation) and cleaning (suppression of tree branches and other vegetal) must be implemented. Secondly, routine surveillance and control of the roof gutters by the Vector Control Agencies are needed. Lastly, the presence of *Ae. aegypti* in the roof gutters must also be included in the information directed towards community participation, in particular when dengue transmission is increasing or during dengue epidemics.

Dengue viruses' circulation in Guadeloupe has dramatically increased during the past years, and at the same time urbanization is developing without any regards to disease transmission. Studies on the relation between housing, vector development, diseases and community involvement are strongly requested to better struggle against this mosquito-transmitted disease.

## Figures and Tables

**Figure 1:**
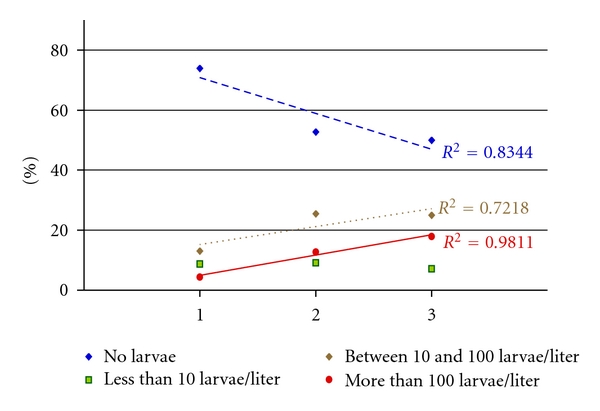
Graphical representation of the abundance of *Ae. aegypti* larvae found in the roof gutters according to the type of water during investigations carried out in Guadeloupe between January and May 2002. (Horizontal axis: 1 = clear water, 2 = water with sediments, 3 = water with vegetal detritus).
